# Editorial: Incorporation of reporting and data systems into cancer radiology

**DOI:** 10.3389/fonc.2023.1171171

**Published:** 2023-03-17

**Authors:** Pilar López-Larrubia, Antonella Santone

**Affiliations:** ^1^ Instituto de Investigaciones Biomédicas Alberto Sols, CSIC-UAM, Madrid, Spain; ^2^ Department of Medicine and Health Sciences “Vincenzo Tiberio”, University of Molise, Campobasso, Italy

**Keywords:** reporting and data system, cancer imaging, magnetic resonance imaging, ultrasound, computed tomography

The potential benefits of Reporting and Data Systems (RADS) are well known: improve the communication between the radiologists and physicians; reduce the omission of relevant information in reports; reduce the variability and errors in image interpretation; facilitate the monitoring of results and provide a tool to ensure quality and research. Furthermore, the slightest error in the interpretation of the legend describing the results of an imaging study can modify both the medical decision to start or not a treatment, and to do it with a more or less invasive technique.

In this sense, for example, the American College of Radiology (ACR) started the development of the Breast Imaging Reporting and Data System (BI-RADS) in 1993. In recent years, several systems have been developed to diagnose other malignancies based on BI-RADS. However, its utility has yet to be demonstrated which is why scoring systems are continually being revised to apply them to daily clinical practice. The RADS systems developed so far are specially designed for breast, colorectal, gynecological, liver, lung, prostate and thyroid cancers.

These aspects are clearly reflected in the research papers accepted in the “*Incorporation of Reporting and Data Systems into Cancer Radiology*” Research Topic. These manuscripts demonstrate how RADS benefit cancer patients, and how improvements to these systems can further benefit patients and optimize disease outcomes. For instance, in Wang et al., the authors investigated the value of contrast-enhanced ultrasound in the differential diagnosis and risk stratification of ACR TI-RADS category 4 and 5 thyroid nodules with non-hypovascular, establishing a risk score with ability to improve both aspects.

In Sun et al., it was evaluated the value of multiparametric magnetic resonance imaging (MIR) including synthetic MRI, diffusion-weighted imaging, dynamic contrast enhanced MRI, and clinical features in breast imaging–reporting and data system (BI-RADS) 4 lesions, and develop an efficient method to help patients avoid unnecessary biopsy.

In Zhou et al., the authors established a predictive model incorporating clinical features and contrast enhanced ultrasound liver imaging- reporting and data system (CEUS LI-RADS) to improve the prediction of microvascular invasion (MVI) in hepatocellular carcinoma (HCC) patients.

In Singh et al., to minimize inter-radiologist variability, the diagnostic performance of an in-house developed semi-automated model using machine learning methods for prostate imaging-reporting and data system version 2.1 (PI-RADS v2.1) scoring was evaluated in prostate cancer patients. The authors in Meng et al., assessed the value of using quantitative parameters from synthetic magnetic resonance imaging (SyMRI) and the Kaiser score (KS) to differentiate benign and malignant breast lesions in patients, identifying that the incorporation of T_1_ values improves the sensitivity and specificity of the KS protocol alone.

In Yang et al., a retrospective study was performed to create a predictive machine learning model based on liver imaging and reporting and data system (LI-RADS) features to identify microvascular invasion in hepatocellular carcinoma (HCC) patients, concluding that LI-RADS may help optimize the management of these patients.

Finally, in Wu et al., the authors conducted a retrospective Chinese population-based study of patients screened for lung cancer by computed tomography to assess the correlation between the probability of lung cancer and the number of lung nodules, concluding that this probability does not change with the number but does change with the size of the nodules.

## Author contributions

All authors listed have made a substantial, direct, and intellectual contribution to the work and approved it for publication.

